# Caretaker-reported quality of life, functionality, and complications associated with assistive mobility cart use in companion animals

**DOI:** 10.3389/fvets.2024.1466405

**Published:** 2024-10-28

**Authors:** Melissa Narum, Erin Miscioscia, Jennifer Repac

**Affiliations:** Department of Comparative, Diagnostic, and Population Medicine, College of Veterinary Medicine, University of Florida, Gainesville, FL, United States

**Keywords:** cart, wheelchair, mobility, assistive device, veterinary rehabilitation, spinal cord injury

## Abstract

**Objective:**

To evaluate the impact of assistive mobility carts on companion animals and caretakers’ quality of life by investigating factors pertaining to caretaker satisfaction, the ability to perform daily tasks, and complication rates.

**Materials and methods:**

A 23-question survey was distributed to caretakers of animals using carts to evaluate the animal and caretakers’ quality of life, acceptance, ability to complete functional tasks, and complications. Data from canine, feline, and rabbit responses were analyzed separately.

**Results:**

Dogs and cats had improved quality of life in 62 and 57% of responses and 61 and 60% for their caretakers, respectively. There was no improvement in the quality of life of rabbits or their caretakers. Regarding the complication rate, 64% were reported to have at least one complication associated with cart use, 53% of which were wounds. Across all species, there was a reported improvement in ability to perform daily tasks and activities.

**Conclusions and clinical relevance:**

Caretakers reported that assistive mobility carts improve both companion animals’ and caretakers’ quality of life, despite high prevalence of complications, including wounds. Future studies exploring specific disease conditions and long-term outcomes will be useful for guiding clinical recommendations.

## 1 Introduction

Assistive mobility devices are designed to improve quality of life by providing independent mobility to the user. In human medicine, assistive devices such as wheelchairs, crutches, and walking canes can be used to aid mobility (1–3). Similarly, assistive mobility devices can be used for companion animals with a range of mobility disorders (4–7). In veterinary medicine, studies have explored the application, acceptance rate, and complications of prosthetic and orthotic assistive mobility devices (8–10). Independent mobility will impact both the animal and caretaker quality of life and can impact the strength of the human-animal bond (11–14).

Veterinary assistive mobility carts, sometimes called “wheelchairs” or “carts,” are generally composed of a saddle or harness attached to a rigid structure supported by 2–4 wheels, depending on individual needs. Numerous brands of veterinary carts offer customized and standard options designed to support animals with mobility disorders. The most common indications for cart use in companion animals are neurological or orthopedic diseases. Spinal cord injury or degenerative conditions are the most common neurological causes leading to impairment or inability to ambulate independently (14, 15). These can include specific conditions, such as intervertebral disc disease and non-compressive myelopathies (such as fibrocartilaginous embolism or acute non-compressive nucleus pulposus extrusion), or degenerative diseases, such as degenerative myelopathy (6, 16). Orthopedic conditions such as joint disease or amputations can also impair an animal’s independent mobility (17, 18).

To the authors’ knowledge, no studies have examined the use of assistive mobility carts in veterinary medicine. Given the growing prevalence of these products, there is a need for research to guide clinical recommendations. The objective of this caretaker survey study is to evaluate how assistive mobility carts impact the quality of life of both companion animals and their caretakers. The secondary aim was to report other factors that may impact overall satisfaction, including cart type and complication rate. We hypothesized that the majority of animals using carts, and their caretakers, will experience improved quality of life.

## 2 Materials and methods

A 23-question online survey was developed to obtain information about assistive mobility cart use, acceptance, and impact on quality of life for both animals and caretakers. This survey was active from 2/1/23 to 2/15/23. The survey was developed on the Qualtrics platform (Qualtrics, Provo, UT) for distribution purposes. The collected information included species, age, cart brand, time to acceptance and daily use, complications and wounds, ability to perform basic tasks/activities, perceived animal and caretaker quality of life and whether use would be recommended to another caretaker. The information was caretaker-reported and anonymized. The survey was designed as a single assessment. The styles of questions included multiple choice, yes/no and select all that apply. There were a couple questions that included an option for a fill-in-the blank response. The survey questions are listed in Supplementary Table 1. The survey was distributed with an introductory paragraph to explain the goals of the survey and reach the appropriate audience. Participation in the survey was intended to be anonymous with the primary requirement being ownership of an animal that previously used or is currently using an assistive mobility cart. The summaries used prior to distribution and at the time of distribution are available in Supplementary Table 1.

### 2.1 Survey distribution

The survey was emailed to the caretakers of companion animals utilizing carts within the University of Florida College of Veterinary Medicine Integrative and Mobility Medicine Service and the American Association of Rehabilitation Veterinarians (AARV), Academy of Physical Rehabilitation Veterinary Technicians (APRVT), and Veterinary Sports Medicine and Rehabilitation (VSMR) listservs. The survey link was also posted on Facebook groups related to canine neurological or orthopedic diseases, the VSMR newsletter, and the VSMR resident Facebook page.

Responses from canine, feline, and rabbit use of carts were analyzed in this study. Responses in a different language or responses flagged as a “bot” response by the Qualtrics security screening were excluded. Responses for which the species was listed as “other” and written text responses were not provided were also excluded. If a specific dog breed was listed for those who selected “other,” these were reorganized appropriately to be counted as “dog” responses. Age was collected in years and grouped into 4 categories: “<1 years old,” “1–6 years old,” “>6 years old” and “deceased.” These age ranges were derived from the AAHA Canine Life Stage Guidelines (19).

### 2.2 Statistics

Survey responses were summarized as counts and binomial proportions as appropriate. For individual proportions, a chi square test of equal proportions between (i.e., positive/negative, yes/no) responses was used and binomial 95% confidence intervals are given. For comparison of differences in proportions between multiple groups a logistic linear model was used. When global tests of grouped differences were found to be significant, *post hoc* group comparisons were made using Tukey’s multiple comparisons procedure with letter groupings and overall significance based on alpha level 0.05.

## 3 Results

A total of 1,778 survey responses were received. Following the application of exclusion criteria, 1,221 survey responses were available for review. There were a total of 954 responses for dogs, 219 for cats, and 46 for rabbits. A portion of responses were incomplete with the completed portions being retained for analysis.

Approximately 42% of all responses listed a neurological cause and 47% listed an orthopedic cause as the reason for cart use. The remaining responses cited either a combination of neurological and orthopedic diseases, unspecified congenital disease, or unknown reasons. Eleven commercial brands of assistive mobility carts were reported to be used in addition to homemade carts. Table 1 displays the number of responses for the six most common brands.^1^^,^ ^2^^,^ ^3^^,^ ^4^^,^ ^5^^,^ ^6^ For dogs, there was an inverse relationship between size and ease of cart loading. As size increased, placement became more difficult (*p* < 0.001).

**Table 1:** Distribution of responses for the 6 most common brands of assistive mobility cart (see text footnotes 1–6).

Brand of cart	Total number responses
Brand 1	248
Brand 2	363
Brand 3	195
Brand 4	172
Brand 5	82
Brand 6	79

### 3.1 Quality of life

For dogs and cats, there was a significant improvement in the quality of life of both animals and caretakers. Dogs were reported to have an improved quality of life in 62% of responses (*p* < 0.001) for animal quality of life and 61% for caretaker quality of life. Cats were reported to have improved quality of life in 57% of responses (*p* = 0.035) and 60% for their caretakers (*p* = 0.0028). For rabbits, there was not a majority response to improvement in quality of life for either the animal (35%, *p* = 0.04) or the caretaker (39%, *p* = 0.14). The specific values are listed in Table 2.

**Table 2:** Impact of assistive mobility cart use on animal and caretaker quality of life (QOL) by species.

	Species	Positive responses	Total responses	*p* value
Animal QOL	All species	715 (60%)	1,195	<0.001
	Dog	571 (62%)	922	<0.001
	Cat	124 (57%)	217	0.035
	Rabbit	16 (35%)	46	0.038
Caretaker QOL	All species	713 (60%)	1,192	<0.001
	Dog	561 (61%)	920	<0.001
	Cat	130 (60%)	216	0.0028
	Rabbit	18 (39%)	46	0.14

The quality of life responses related to the type of cart and species of animals and caretakers are detailed in Table 3. For dogs and cats, there was a statistically significant improvement in quality of life for both the animal and caretaker when using both quad carts (4 wheels) and hind wheel carts, but not front wheel carts. For dogs, use of hind wheel carts had a statistically significant improvement when compared to quad or front wheel carts for animal quality of life. In cats, quad carts had a statistically significant improvement in animal quality of life when compared to front wheel carts. There was no statistical difference between hind wheel carts and either quad or front wheel carts for cats. When considering caretaker quality of life, there was a statistically significant improvement in quality of life for both quad carts and hind wheel carts when compared to front wheel carts in both dogs and cats. There was no statistical difference between quad carts and hind wheel carts for either of these species.

**Table 3:** Cart type impact (quad, front wheel, hind wheel) on animal and caretaker quality of life (QOL) for each species.

	Species	Quad (positive/total)	Front wheel (positive/total)	Hind wheel (positive/total)	*p* value
Animal QOL	Dog	140/260 (54%)^b^	105/223 (47%)^b^	323/435 (74%)^a^	<0.001
	Cat	59/88 (67%)^a^	41/89 (46%)^b^	24/38 (63%)^ab^	0.014
	Rabbit	3/11 (27%)	7/21 (33%)	5/13 (39%)	0.85
Caretaker QOL	Dog	143/260 (55%)^b^	111/223 (50%)^b^	304/433 (70%)^a^	<0.001
	Cat	59/87 (68%)^a^	42/89 (47%)^b^	28/38 (63%)^a^	0.0035
	Rabbit	6/11 (55%)	7/21 (33%)	5/13 (39%)	0.51

Percentages with different superscripts (eg. ab) indicate statistical differences between the columns. Rabbit responses were not included in the Tukey–Kramer least square means comparison.

There was no difference in the reported quality of life between cart types in rabbits (*p* = 0.51). There was no significant relationship between the duration of cart use per day and quality of life for animals or caretakers across all species. There was a direct relationship between animal size and both positive animal and caretaker quality of life (*p* < 0.001).

Across all species, the majority of caretakers (79%) were likely to recommend cart use to others. Caretakers who reported improved animal (89%; *p* < 0.001) and caretaker (91%; *p* < 0.001) quality of life were more likely to recommend cart use to others.

### 3.2 Complications

The overall complication rates according to the species are listed in Table 4. Across all species, 64% reported complications, and 53% of the complications were wounds. Animals fitted by a veterinarian had a higher reported complication rate (72%) than those not fitted by a veterinarian (47%; *p* < 0.001). There was no association between age of animal and complication rate across all species.

**Table 4:** Complication rates associated with assistive mobility cart use by species.

Species	Responses reporting complications	Total responses	Complication rate (%), [CI 95%]	*p* value
All species	782	1,230	64% [61, 66%]	<0.001
Dog	575	954	60% [57, 63%]	<0.001
Cat	166	219	76% [70, 81%]	<0.001
Rabbit	33	46	72% [59, 85%]	0.0032

**Figure 1:**
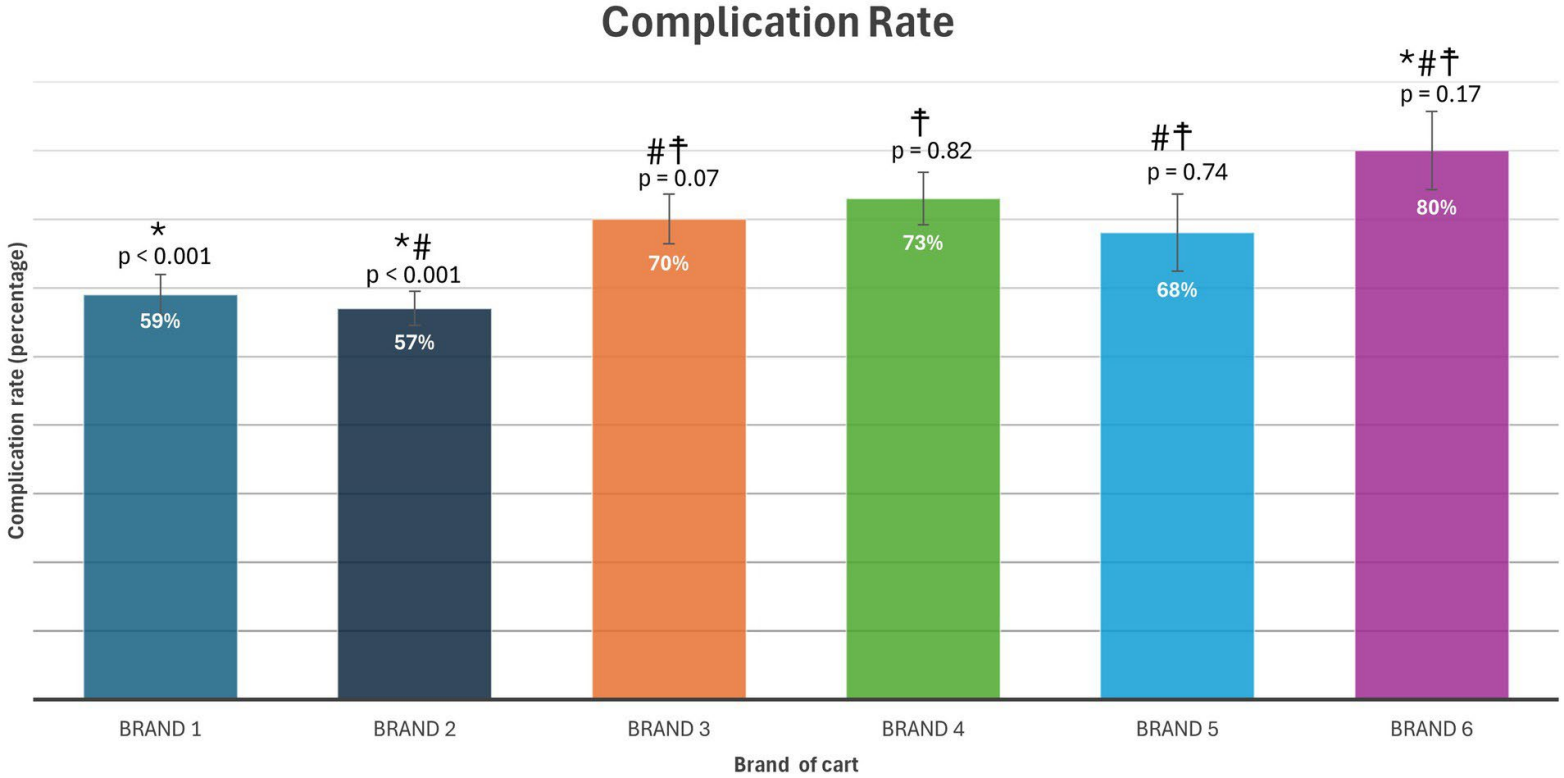
Complication rate for six most common brands (see text footnotes 1–6) with Tukey grouping of significance. Percentages with different symbols are statistically different between brands (*p* < 0.05).

Across all cart brands, the complication rate was greater than 50%. The complication rate was significantly lower for Brands 1 and 2 compared to the remaining brands (*p* < 0.001). The specific percentages of survey response reporting complications for the six most common brands are reported on Figure 1. There was no significant relationship between the location of the wounds and the cart brand (*p* = 0.05).

The most common location for wounds to develop included the “inside of hind upper leg or thigh” followed by the “inside of upper front leg or armpit” region and “top of paws/foot.” The specific locations of the wounds are summarized in Table 5.

**Table 5:** Location of wounds associated with assistive mobility cart use.

Location	Number of wounds	Percentage of total wounds
Top of paws/foot	87	14%
Inside of hind upper leg/thigh	175	28%
Inside of upper front leg/armpit	136	22%
Belly	76	12%
Back	62	10%
Tail	39	6%
Head or Neck	42	7%
Back of ankle/hock	1	0.2%
Other	3	0.5%
Total number of wounds	621	

### 3.3 Functional tasks

Most animals showed improved functionality when they used carts. Across all species, a higher percentage of animals were reported to have an easier time performing the functional tasks, apart from the ability to rest or sleep which was only improved for cats and rabbits. Improvement in the ability to play had the highest positive response rate across all species. Table 6 outlines the results of functional task performance across all species. There was no association between time of use per day and likelihood for a caretaker to recommend cart use to another caretaker across all species.

**Table 6:** Improvement in functional task performance across species using assistive mobility carts.

Task	Species	Yes	Total	Yes responses (%), [CI 95%]
Play	Dog	723	919	79% [76, 81%]
	Cat	177	216	82% [77, 87%]
	Rabbit	32	46	70% [56, 83%]
Eat/drink	Dog	732	918	80% [77, 82%]
	Cat	152	216	70% [64, 76%]
	Rabbit	31	46	67% [54, 81%]
Urinate	Dog	525	914	57% [54, 61%]
	Cat	140	216	65% [58, 71%]
	Rabbit	30	46	65% [51, 79%]
Defecate	Dog	493	913	54% [51, 57%]
	Cat	140	215	65% [59, 71%]
	Rabbit	27	46	61% [47, 75%]
Walk/run	Dog	676	914	74% [71, 77%]
	Cat	148	215	69% [63, 75%]
	Rabbit	24	46	52% [38, 67%]
Rest/sleep	Dog	440	914	48% [45, 51%]
	Cat	158	214	74% [68, 80%]
	Rabbit	37	46	80% [69, 92%]

## 4 Discussion

In this study, the use of assistive mobility carts resulted in a perceived improvement in the quality of life of dogs and cats, as well as their caretakers. However, the use of rabbit carts did not improve the quality of life of animals or caretakers. In general, the goal of using an assistive mobility device is to improve independent mobility and interaction with the environment (2). Within animal ownership and veterinary medicine, there is an inherent dependence between an animal and its caretaker to meet basic needs (7, 11). Carts can improve functional independence, thereby alleviating the caregiver burden.

If indicated, carts may be used as a temporary aid in recovery or lifelong. Carts can be used as a part of the rehabilitation process to help keep animals in a standing position, especially when they are too weak to maintain this posture (20). As animals rebuild strength and coordination, cart use can be phased out, particularly in neurological rehabilitation. Additionally, for animals with severe spinal cord injuries, carts can assist in the development of reflexive walking by supporting the animal’s weight along with other gait retraining physical rehabilitation activities (21–23). However, the use of carts remains a controversial topic. In Sweden, the use of assistive mobility carts is illegal because of the ethical concerns associated with non-ambulatory animals (13). It is not permissible to use carts, even temporarily, to assist ambulation. Further studies are necessary to determine which conditions would benefit the most from assistive mobility carts.

For humans, a variety of assistive devices can be used to improve independent mobility and comfort. Wheelchairs, walkers, and canes are analogous to assistive mobility carts for companion animals. In one study looking at adults with late-life disability, 87% of respondents stated that their quality of life was “fair,” “good” or “very good” and that sense of control and dignity had the largest influence on their quality of life (24). Quality of life was also dependent on the acceptance of disability and a shift in focus to functionality, as opposed to limitations. Further research has shown that electric-powered chairs improve mobility and comfort for severely disabled people, but not independence or social interaction (3). Another study found that the use of assistive devices contributes to socioeconomic interaction, independence, and self-esteem, which are important factors for dignity (1). Challenges with assistive devices include maintenance access, infrastructure, costs, and ignorance. The stigma of using the device in public or at a place of occupation is one of the most prevalent psychological barriers (25). Assistive mobility carts may also have similar impacts on the quality of life, as seen in this study, and animals may face similar barriers to access.

Owners can be hesitant to consider carts out of concern that their pets will be unable to perform basic functions and thus have a poor quality of life. However, in this study, owners reported carts allowed animals to better perform 3 out of 4 Basic Activities for Independent Mobility (BADIM) and 4 out of 7 Instrumental Activities for Daily Quality of Life (IADQOL) described by Frye et al. (20). In addition to facilitating independent mobility, play, and eating and drinking, carts also enable animals to better posture for urination and defecation. Carts keep animals elevated during elimination and during bladder expression of incontinent animals (6, 26). In this study, the majority of caretakers stated they would recommend a cart to other caretakers, indicating the perceived value of this assistive device.

Fewer dogs had improvement in the ability to sleep or rest when using the cart compared to those who showed no improvement. The cart is designed to support a standing posture, and a completely sternal, or resting, posture is not physically possible during use. Cart use requires a caretaker to place the animal both in and out of the cart and supervise use to avoid fatigue or injury. Interestingly, responses for cats and rabbits reported overall improved ability for the animal to sleep or rest while in the cart, and this may be due to differences in size, flexibility, or conformation compared to dogs. Further observational studies are needed to better understand differences in the performance of functional tasks across companion animal species.

In terms of quality of life, there was improvement for both animals’ and caretakers’ quality of life for both quad and hind wheel carts. There was not a reported improvement in quality of life for either the animal or caretaker across all species for front wheel cart users. This may be related to differences in weight-bearing between the forelimbs and hindlimbs. In healthy dogs, the forelimbs bear approximately 60% of the body weight and the hindlimbs bear approximately 40% (27). For this reason, it may be easier for animals to acclimate to a hindlimb cart. For quad carts, it is possible that having the animals supported into a standing posture can make them more interactive with their surroundings and able to move with assistance or independently which would lead to improved quality of life. We suspect large dogs had the most improved animal and caretaker quality of life due to the alleviation of the greater physical burden of carrying a larger dog compared to smaller breeds. Many large breed dogs may appreciate the independence of cart activity versus a small breed dog who may be accustomed to being carried.

All assistive devices carry a risk of complications and failure of acceptance. In studies investigating veterinary prosthetic or orthotic use, skin sores, device failure or poor acceptance or compliance are commonly reported (8, 9, 28, 29). Behavioral or compliance issues may arise if the animal does not want to use the device or if the caretaker is unable or unwilling to assist the animal into the device. Specifically trained veterinary personnel with knowledge of assistive mobility carts may be used to alleviate acceptance or compliance issues.

The overall complication rate (64%) in this study was similar or lower when compared to what is reported in veterinary prosthetic and orthotic literature. In a study on socket prostheses, the short and long-term complication rates were 62 and 19%, respectively (9). In another study on both orthoses and prostheses, 91% of patients experienced at least one complication (8). The most common complications cited were skin complications, mechanical issues and non-acceptance by the patient.

In the current study, the most common location for wounds to develop was the inner thigh, followed by the axillary region and the tops of the paw or feet. This result is likely due to contact with the supporting saddle, harness of the cart and contact with the ground. The saddles are generally constructed out of rubber or other firm materials. In addition, many animals using carts are incontinent, posing a greater challenge in maintaining skin hygiene. Further research into the use of different materials to line these areas is needed to help reduce wound development in these high-contact areas. A high prevalence of wounds forming on the tops of the paws is suspected to be due to the dragging of the paw on abrasive flooring. Paw wounds can be prevented by applying protective footwear or sling supports to prevent foot contact with the floor. Species variation in terms of skin thickness or fur type can contribute to formation of wounds. Specific carts are generally designed for a specific species of animal which can also impact the overall fit and lead to wounds or other complications. Further studies looking into these variables are needed to better understand the impact on wound development.

We were surprised to find that dogs who were fitted by a veterinarian experienced a higher incidence of complications. This may in part be due to selection bias; more challenging cases may be more likely to present for veterinary care. Another consideration is the variability of veterinary training. Rehabilitation is not included in the core veterinary curriculum of most veterinary schools, and thus, veterinarians generally lack exposure to assistive devices. Training programs range from rehabilitation certification to board certification (Diplomate of the American College of Veterinary Sports Medicine and Rehabilitation). Even with advanced rehabilitation training, assistive mobility cart fitting education is not standardized. Moreover, veterinarians often only observe animals in a clinic setting, limiting the ability to troubleshoot acclimatization and fitting challenges that may occur exclusively in the home environment.

Positive reinforcement and physical rehabilitation focused on the appropriate device use are generally recommended to improve success. Behavioral acclimation to ensure cart acceptance is critical, especially in cases when a cart is the only way an animal can ambulate independently (4). Future prospective studies investigating the impact of rehabilitation programs guiding cart use are warranted.

There were several limitations inherent to the survey-based nature of this study. Information was self-reported by caretakers and medical indications were not confirmed via medical records. This can especially impact the reason for use and complication variables. There could have been a selection bias of respondents based on their experiences with cart use. Caretakers with very good or very poor experiences may be more likely to participate in the survey. Additionally, incentivized surveys can be susceptible to spurious or “bot” responses. To limit this possibility, a “bot” response detection service from the Qualtrics platform was applied to filter sham responses. Survey question interpretation was dependent on the participant and may have been variable. This may have influenced responses and may have differed from the author’s intended goal of the question. The single-use survey format may have excluded additional data from animals that have used multiple brands or types of carts. The use of binary response (yes/no) questions may have oversimplified more nuanced answers and should be avoided in future surveys to avoid leading questions. Another limitation was not requesting the training levels of the veterinarians involved in the cart fitting. Future studies are required to investigate the impact of how guidance from a board-certified veterinary sports medicine and rehabilitation specialist impacts cart complication and acceptance rates.

## 5 Conclusion

Based on this survey study, assistive mobility carts improved the quality of life of dogs and cats with mobility disorders and of their caretakers. There was no improvement in quality of life for the majority of rabbits or their caretakers. Carts are generally well-accepted and facilitate activities of daily living. Similar to veterinary orthotics and prosthetics, wounds are the most commonly reported complication. Future studies exploring the impact on patient outcomes and factors influencing success, acceptance and complication rates are needed to guide clinical recommendations.

## Data Availability

The original contributions presented in the study are included in the article/Supplementary material, further inquiries can be directed to the corresponding author.

## Footnotes

^1^Walkin’ Pets, 105 Route 101A, Suite 18, Amherst, NH 03031.

^2^Eddie’s Wheels, Eddie’s Wheels Custom Dog Wheelchairs 140 State Street, Shelburne Falls, MA 01370.

^3^K9 Carts, Paw Prosper Company, 2,851 Placida Rd., Units A & B Englewood, FL 34224.

^4^Doggon’ Wheels, Doggon’ Wheels LLC, 1032 Irving St #501, San Francisco, CA 94116.

^5^Ruff Rollin’, 1,505 14th Street SW, Suite #103, Great Falls MT 59404.

^6^Best Friend Mobility, 279 Hwy 57 S STE4, Little River, SC 29566, USA.

## References

[1] Borade N Ingle A Nagarkar A. Lived experiences of people with mobility-related disability using assistive devices. Disabil Rehabil Assist Technol. (2021) 16:730–4. doi: 10.1080/17483107.2019.1701105, PMID: 31833435

[2] Cowan Fregly BJ Boninger ML Chan L Rodgers MM Reinkensmeyer DJ. Recent trends in assistive technology for mobility. J Neuroeng Rehabil. (2012) 9:20. doi: 10.1186/1743-0003-9-20, PMID: 22520500 PMC3474161

[3] Davies A De Souza LH Frank AO. Changes in the quality of life in severely disabled people following provision of powered indoor/outdoor chairs. Disabil Rehabil. (2003) 25:286–90. doi: 10.1080/0963828021000043734, PMID: 12623619

[4] Magidenko SR Peterson NW Pisano G Buote NJ. Analysis of patient outcome and owner satisfaction with double limb amputations: 14 dogs and four cats. J Am Vet Med Assoc. (2022) 260:884–91. doi: 10.2460/javma.21.04.0199, PMID: 35333746

[5] Marcellin-Little DJ Drum MG Levine D McDonald SS. Orthoses and exoprostheses for companion animals. Vet Clin North Am Small Anim Pract. (2015) 45:167–83. doi: 10.1016/j.cvsm.2014.09.009, PMID: 25432685

[6] Olby NJ Moore SA Brisson B Fenn J Flegel T Kortz G . ACVIM consensus statement on diagnosis and management of acute canine thoracolumbar intervertebral disc extrusion. J Vet Intern Med. (2022) 36:1570–96. doi: 10.1111/jvim.16480, PMID: 35880267 PMC9511077

[7] Sedlacek J Rychel J Giuffrida M Wright B. Nonsurgical rehabilitation in dachshunds with T3-L3 myelopathy: prognosis and rates of recurrence. Front. Vet. Sci. (2022) 9:934789. doi: 10.3389/fvets.2022.934789, PMID: 35928109 PMC9343690

[8] Rosen S Duerr FM Elam LH. Prospective evaluation of complications associated with orthosis and prosthesis use in canine patients. Front. Vet. Sci. (2022) 9:892662. doi: 10.3389/fvets.2022.892662, PMID: 35967994 PMC9372342

[9] Wendland TM Seguin B Duerr FM. Retrospective multi-center analysis of canine socket prostheses for partial limbs. Front. Vet. Sci. (2019) 6:100. doi: 10.3389/fvets.2019.00100 31024938 PMC6460115

[10] Wendland TM Seguin B Duerr FM. Prospective evaluation of canine partial limb amputation with socket prostheses. Vet. Med. Sci. (2023) 9:1521–33. doi: 10.1002/vms3.1146, PMID: 37287388 PMC10357256

[11] Freeman PM Holmes MA Jeffery ND Granger N. Time requirement and effect on owners of home-based management of dogs with severe chronic spinal cord injury. J Vet Behav. (2013) 8:439–43. doi: 10.1016/j.jveb.2013.06.001

[12] Lee S Wendland TM Rao S Magee C. Orthotic device use in canine patients: owner perception of quality of life for owners and patients. Front. Vet. Sci. (2021) 8:709364. doi: 10.3389/fvets.2021.709364, PMID: 34805329 PMC8600258

[13] Mojarradi A De Decker S Bäckström C Bergknut N. Safety of early postoperative hydrotherapy in dogs undergoing thoracolumbar hemilaminectomy. J Small Anim Pract. (2021) 62:1062–9. doi: 10.1111/jsap.13412, PMID: 34423457

[14] Olby NJ da Costa RC Levine JM Stein VM and the Canine Spinal Cord Injury Consortium (CANSORT SCI). Prognostic factors in canine acute intervertebral disc disease. Front Vet Sci. (2020) 7:596059. doi: 10.3389/fvets.2020.596059, PMID: 33324703 PMC7725764

[15] Rossi G Stachel A Lynch AM Olby NJ. Intervertebral disc disease and aortic thromboembolism are the most common causes of acute paralysis in dogs and cats presenting to an emergency clinic. Vet Rec. (2020) 187:e81. doi: 10.1136/vr.105844, PMID: 32471959

[16] Kobatake Y Nakata K Sakai H Sasaki J Yamato O Takashima S . The long-term clinical course of canine degenerative myelopathy and therapeutic potential of curcumin. Vet Sci. (2021) 8:192. doi: 10.3390/vetsci8090192, PMID: 34564586 PMC8471773

[17] Mich PM. The emerging role of veterinary orthotics and prosthetics (V-OP) in small animal rehabilitation and pain management. Top Companion Anim Med. (2014) 29:10–9. doi: 10.1053/j.tcam.2014.04.002, PMID: 25103884

[18] Wright A Amodie DM Cernicchiaro N Lascelles BDX Pavlock AM Roberts C . Identification of canine osteoarthritis using an owner-reported questionnaire and treatment monitoring using functional mobility tests. J Small Anim Pract. (2022) 63:609–18. doi: 10.1111/jsap.13500, PMID: 35385129 PMC9543207

[19] Creevy K Grady J Little S Moore G Strickler B Thompson S . 2019 AAHA Canine Life Stage Guidelines. J Am Anim Hosp Assoc. (2019) 55:267–90. doi: 10.5326/JAAHA-MS-6999, PMID: 31622127

[20] Frye C Carr BJ Lenfest M Miller A. Canine geriatric rehabilitation: considerations and strategies for assessment, functional scoring, and follow up. Front. Vet. Sci. (2022) 9:842458. doi: 10.3389/fvets.2022.842458, PMID: 35280131 PMC8914307

[21] Gallucci A Dragone L Menchetti M Gagliardo T Pietra M Cardinali M . Acquisition of Involuntary Spinal Locomotion (spinal walking) in dogs with irreversible thoracolumbar spinal cord lesion: 81 dogs. J Vet Intern Med. (2017) 31:492–7. doi: 10.1111/jvim.14651, PMID: 28238221 PMC5354022

[22] Lewis MJ Jeffery ND Olby NJ the Canine Spinal Cord Injury Consortium (CANSORT-SCI). Ambulation in dogs with absent pain perception after acute thoracolumbar spinal cord injury. Front. Vet. Sci. (2020) 7:560. doi: 10.3389/fvets.2020.00560, PMID: 33062648 PMC7479830

[23] Martins Â Silva CM Silva CM Gouveia D Cardoso A Coelho T . Spinal locomotion in cats following spinal cord injury: a prospective study. Animals. (2021) 11:1994. doi: 10.3390/ani11071994, PMID: 34359122 PMC8300158

[24] King J Yourman L Ahalt C Eng C Knight SJ Pérez-Stable EJ . Quality of life in late-life disability: "I don't feel bitter because I am in a wheelchair". J Am Geriatr Soc. (2012) 60:569–76. doi: 10.1111/j.1532-5415.2011.03844.x, PMID: 22288767 PMC3619719

[25] Saia T Vogel E Salazar S. “We need a world we can operate in”: exploring the relationship between societal stigma and depression among wheelchair users. Disabil Health J. (2024) 17:101624. doi: 10.1016/j.dhjo.2024.101624, PMID: 38631970

[26] Jeffery N Levine J Olby N Stein V. Intervertebral disk degeneration in dogs: consequences, diagnosis, treatment, and future directions. J Vet Intern Med. (2013) 27:1318–33. doi: 10.1111/jvim.12183, PMID: 24010573

[27] Bockstahler BA Skalicky M Peham C Müller M Lorinson D. Reliability of ground reaction forces measured on a treadmill system in healthy dogs. Vet J. (2007) 173:373–8. doi: 10.1016/j.tvjl.2005.10.004, PMID: 16324859

[28] Carr BJ Canapp S Petrovitch JL Campana D Canapp D Leasure CS. Retrospective study on external canine limb prosthesis used in 24 patients. Vet Evid. (2018) 3:1–13. doi: 10.18849/ve.v3i1.118

[29] Phillips A Kulendra E Bishop E Monk M Parsons K House A. Clinical outcome and complications of thoracic and pelvic limb stump and socket prostheses. Vet Comp Orthop Traumatol. (2017) 30:265–71. doi: 10.3415/VCOT-16-09-0127, PMID: 28636059

