# Correction: Caretaker-reported quality of life, functionality, and complications associated with assistive mobility cart use in companion animals

**DOI:** 10.3389/fvets.2026.1846252

**Published:** 2026-04-24

**Authors:** Frontiers Production Office

**Affiliations:** Frontiers Media SA, Lausanne, Switzerland

**Keywords:** cart, wheelchair, mobility, assistive device, veterinary rehabilitation, spinal cord injury

When the article was published, Supplementary files **Supplementary Data Sheet 1** and **Supplementary Table 1** were omitted. The files have now been published.

The original version of this article has been updated.

