# Supplementary material for: Caretaker-reported quality of life, functionality, and complications associated with assistive mobility cart use in companion animals
Source: Front Vet Sci. 2024 Oct 28;11:1466405. doi: 10.3389/fvets.2024.1466405 (PMC11551849; doi:10.3389/fvets.2024.1466405)
Supplement: Supplementary file 1 [file Table_1.docx]

**Supplementary Table 1:** Survey questionnaire for assessing assistive mobility cart use in companion animals.

The University of Florida College of Veterinary Medicine is conducting an anonymous survey study to determine if use of assistive mobility carts impacts animal and caretaker quality of life. Eligible participants include owners who have or have previously had a pet that used an assistive mobility cart. Participants can select the option to anonymously be entered in a raffle to win a $50 Amazon gift card following survey completion. Please look for a second message containing the link to the survey to be released within the next week.”

We are ready to start our cart survey, I will send you the information to post and the link. Thank you for helping us with this survey!

The University of Florida College of Veterinary Medicine is conducting an anonymous survey study to determine if use of assistive mobility carts impacts animal and caretaker quality of life. Eligible participants include owners who have or have previously had a pet that used an assistive mobility cart. Once you have completed the survey, you can select the option to be anonymously entered in a raffle to win a $50 Amazon gift card. Winners will be notified at study completion. This survey will close at 11:45pm ET on 2/15/23. We appreciate your time and participation in this study.

Link to the survey: https://ufl.qualtrics.com/jfe/form/SV_54EO0r3RgwxXIQS

| **Question** | **Responses** |
| --- | --- |
| Question 1: What species is your pet? | - Dog - Cat - Rabbit - Other: ___ |
| Question 2: What size is your pet? | - Small breed dog (<20 lbs) - Medium breed dog (21-49 lbs) - Large breed dog (> 50 lbs) - My pet is not a dog |
| Question 3: How old is your pet (in years)? | - *Text box* |
| Question 4: What type of cart does your pet use? | - Quad cart (4 wheels) - Front wheel only - Hind wheel only |
| Question 5: What brand of cart does your pet use? This may be found on the frame of the cart. | - Walkin Wheels/Handicapped Pets - Eddie’s Wheels - K9 Carts - Doggon Wheels - Ruff Rollin’ - Best Friend - I don’t know - Other: ___ |
| Question 6: Why does your pet use a cart? (Select all that apply) | - Spinal/neurological problem - Orthopedic/joint disease - Amputation - I don’t know - Other: ___ |
| Question 7: Was your cart fitted by a veterinarian? | - Yes - No |
| Question 8: How long did it take for your pet to accept using the cart? | - Immediately - Days - Weeks - Months - N/A: My pet did not accept using the cart |
| Question 9: How long has your pet had a cart? | - Less than 1 month - 1-3 months - 4-12 months - Longer than 12 months |
| Question 10: On average, how long does your pet use the cart (in total) each day? | - Less than 30 mins - 30 mins to 2 hours - More than 2 hours - N/A: my pet does not use their cart |
| Question 11: How easy is it to place your pet in their cart? | - Extremely easy - Somewhat easy - Neither easy nor difficult - Somewhat difficult - Extremely difficult |
| Question 12: What issues have you had with the cart? | - No issues - Wounds - Poor fitting - Mechanical issues - My pet does not like their cart - Other: ___ |
| Question 13: If “wounds” was selected in the prior question, please select the area where the wounds were located. | - Top of paws/foot - Inside of hind upper leg/thigh - Inside of upper front leg/armpit - Belly - Back - Tail - Head or Neck - My pet did not have wounds - Other: ___ |
| Question 14: Is your pet able to play while using their cart? | - Yes - No |
| Question 15: Is your pet able to eat and drink without further assistance while using their cart? | - Yes - No |
| Question 16: Does your pet have an easier time urinating while in their cart compared to not being in their cart? | - Yes - No |
| Question 17: Does your pet have an easier time defecating while using their cart compared to not being in their cart? | - Yes - No |
| Question 18: Does your pet have an easier time walking or running in their cart compared to when they are not in their cart? | - Yes - No |
| Question 19: Is your pet able to rest or sleep comfortably while in their cart? | - Yes - No |
| Question 20: How has the cart impacted YOUR PET'S quality of life? Select a statement that resonates with you: | - Positive - Using a cart for my pet has improved their quality of life - Neutral - Using a cart for my pet has made no difference in their quality of life - Negative - Using a cart for my pet has worsened their quality of life |
| Question 21: How has using a cart for your pet affected YOUR quality of life? Select a statement that resonates with you: | - Positive - Using a cart for my pet has improved my quality of life - Neutral - Using a cart for my pet has made no difference my quality of life - Negative - Using a cart for my pet has worsened my quality of life |
| Question 22: How likely are you to recommend using a cart to another pet owner? | - Extremely likely - Somewhat likely - Neither likely nor unlikely - Somewhat unlikely - Extremely unlikely |
| Question 23: Would you like to enter the raffle to win a $50 Amazon gift card? Winners will be randomly chosen after the survey closes on 2/15/23. | - Yes - No |
